# Time to readmission and associated factors after post treatment discharge of severe acute malnourished under-five children in Pawe General Hospital

**DOI:** 10.1186/s41043-022-00308-8

**Published:** 2022-07-08

**Authors:** Fassikaw Kebede

**Affiliations:** Department of Epidemiology and Biostatics, School of Public Health, College of Health Science, Woldia University, 400 Woldia Town, Ethiopia

**Keywords:** Time to relapse, Children, Malnutrition, Ethiopia

## Abstract

**Background:**

Relapse or repeated episodes is the admission of a child with the diagnosis of severe acute malnutrition (SAM) after being discharged to a status of treated and cured from a stabilizing center. A child may experience more than one episode of SAM depending on the improvement of the underlying comorbidity. Thus, this study aimed to estimate the time to readmission of SAM and associated factors for under-five children in North West Ethiopia.

**Methods:**

An institution-based retrospective cohort study was employed in 760 files of under-five children spanning from 2014/15 to 2019/20. The data extraction tool was developed from SAM treatment guidelines and medical history sheets. Epi Data version 3.2 and STATA version 14 were used for data entry and final analysis, respectively. After checking all assumptions, the multivariable Cox Proportional Hazard model was fitted to the isolated independent predictors for time to readmission. A categorical variable with *p* < 0.05 was considered a risk factor for the relapse of SAM.

**Result:**

The mean (± SD) age of participant children was 27.8 (± 16.5) months with mean (± SD) time to relapse of SAM cases were 30.4(± 21.39) weeks posttreatment discharge. The overall incidence density rate of relapse was determined as 10.8% (95% CI 8.3; 12.6). The average time (± SD) for treatment recovery from the first admission of the SAM case was 28.8(± 18.7) days. Time of readmission was significantly associated with living in rural resident (AHR 5⋅3 = 95% CI, 2⋅95, 13⋅87, *p* = 0.021), having HIV infection (AHR6⋅8 = 95%CI; 4.1–11.9 *p* = 0.001), and first admission with edema (AHR = 3.5 = 95% CI; 1.92, 6.2, *p* = 0.018).

**Conclusion:**

Nearly one in every ten severely acute malnourished under-five children relapsed within a mean time to relapse 30.4(± 21.39) weeks posttreatment discharge. Time to relapse was significantly associated with being a rural resident for children, having edema during the first admission, and being HIV-infected cases. A protocol ought to be drafted for extending Supplementary Nutrition in Acute Malnutrition management program following discharge is highly needed.

**Supplementary Information:**

The online version contains supplementary material available at 10.1186/s41043-022-00308-8.

## Introduction

Severe acute malnutrition (SAM) is defined by < 70% weight for length/height (WFL/H), visible severe wasting, the presence of pitting edema, and in children 6 to 59 months of age, mid-upper arm circumference (MUAC) < 110 mm [1, 2]. SAM is a worldwide problem and one of the top dead diseases for children less than five years of age, and children with SAM have nine times more mortality rates than their peers do have [3]. It is responsible directly or indirectly for 60% of the 10.9 million deaths annually among under-five children, and two-thirds of these deaths occur during the first year of life [4–6]. Globally, in 2018, one in 12 of the estimated 52 million children under five had SAM [7, 8], and 2.9 million of these children were admitted for inpatient treatment [2, 4, 9].

The peak age for SAM is 6–18 months, which is the time of fast growth and brain development [4, 10] and has several immediate, underlying, and fundamental factors [11]. Every month, over 25,000 children with SAM are admitted to Ethiopia, and survivors are more likely to perform poorly in school and, once they are, adults, and girls are more likely to suffer from complications [4]. Early identification of SAM is important for initiating treatment and minimizing the risk of complications, which can be done in both community and healthcare settings using appropriate indicators [10]. It can also be prevented by specific interventions including the promotion of exclusive breastfeeding, vaccination, and timely healthcare-seeking behaviors [12].

Many children younger than 5 years in developing countries are exposed to multiple risks, including poverty, malnutrition, poor health, and not stimulating home environments [6, 9]. These can detrimentally affect their cognitive, motor, and social-emotional development, leading to repeated bouts of SAM [13]. Nearly half of all deaths in under-five children are attributable to undernutrition through increasing the frequency and severity of infections and delaying recovery [14]. Relapse after treatment is also another challenge of SAM cases happening usually nearly 4 months of post-discharge [15]. Some instant research findings indicated that the readmission rate of SAM posttreatment discharge was significantly high. This has been reported as 9% in Bangladesh and 34.6% in Ethiopia. Even though the WHO recommends that children with SAM who are discharged from treatment, the programmer should be periodically monitored to avoid relapse with strong recommendations [1, 4]. However, in Ethiopia, there is a lack of studies that address either time to relapse or post-discharge status [4, 5]. As we have seen above, after treatment of SAM and discharge under five children may face multiple health challenges, so this study is paramount and important for the government to focus on the postdischarge status of SAM children. Thus, this study aimed to estimate the time to readmission of Severe Acute Malnutrition (SAM) and associated risk factors among under-five children admitted in Pawe General Hospital.

## Methods

### Study area, design, and setting

The study was conducted in the Pediatric ward of the Pawe general and referral hospital (PGRH) in the North-Western province of Benishangul Gumuz, which is 560 km from Addis Ababa, the capital city of Ethiopia. According to the 2019 national population projection, this region has an estimated 1.21 million inhabitants. The Pediatric ward has 152 beds and a separate SC center for children with SAM.

### Study design

This was a facility-based retrospective cohort study that was conducted among SAM children under five years who were admitted to Pawe General Hospital, Northwest Ethiopia, from 2015/16 to 2019/2020.

### Sample size determination

The sample size was calculated by using log-rank survival data analysis of the double population proportions formula by using the following assumption proportion formula based on the following important assumptions—95% confidence level, 80% optimum statistical power, and taking one error 5%. Considering a study that was conducted in the same place in northwest Ethiopia and taking rural residents as a predictor variable for the exposed group of under-five children denoted by q1 (0.52) and urban residents not exposed group and denoted by q0 (0.48). After adding 15% incomplete medical records, the final sample size was found 630. Nevertheless, children admitted and started inpatient treatment at the study hospital from 2015/2016 to 2020/202 was found to be 790. Hence, all study participants were included in the study without a sampling procedure.

### Data collection instruments and quality control

A standard and pretested data extraction tools were used to extract the required information from the case notes both for new and readmitted cases. Before the actual data collection, the prepared checklist of variables was pretested in 28 case notes of SAM children from Jawi Primary hospital. The two-day training was given to two diploma nurses and one BSc public health officer on the objective of study outcome and maintaining data confidentiality. An assigned supervisor was strictly followed and oversaw the completeness of the collected data and feedback was given daily.

### Data processing and analysis

After coding, data were entered into Epi Data version 4.2 and then exported to STATA (SE) version-14 for further analysis. Before analysis, the data were cleaned, and simple frequency, cross-tabulation, and categorization of continuous variables were done. Descriptive nonparametric survival analyses such as the life table and Kaplan Meier survival curve were used to estimate the cumulative probability of SAM admitted children and the median time to relapse to readmission, respectively. The Kaplan Meier plot compared the survival times for two or more group categories on the SAM graph to detect a difference in new or readmission cases. Assessing whether or not there is a real statistically significant difference between the two groups will be tested by using the log-rank test. Under the log-rank test, the null hypothesis (there is no difference between the survival times of the two groups) were tested against the alternative that the survival time were not the same among categories, and the stratum of covariates was considered as statistically significant at the *p* value 0.05 in the log-rank test.

Finally, we used *Cox proportional* hazards regression model with robust sandwich covariance matrix estimates to account for repeated measurements for each malnourished child. Before running multiple *Cox proportional regression*, the test of proportionality hazard assumption was checked using graphical methods (*log–log plot*) and statistical methods (*global goodness of fit test*, time-dependent).Variables with *P* value < 0.2 in the bivariable Cox regression analysis were included in the multivariable Cox regression model to determine the factors that caused relapse time to SAM.

## Result

### Socio-demographic characteristics of SAM admitted children

From January 1st, 2015 to December 30, 2020, seven hundred ninety-six (*N* = 796) malnourished under-five children were admitted in Pawe hospital. Of the total children who started treatment, thirty-six (*N* = 36) charts were excluded due to incompleteness. The overall mean (± SD) age of the respondents was found 27.8 (± 16.5) months. The majority 458 (60.26%) of the participants were ≤ 24 months of age. Nearly three to fourth 556(73.16%) of the cases were from rural residents. Concerning breastfeeding status, more than two-thirds of 465(61.18%) under-five children were breastfeeding during the first admission (Table 1).

**Table 1:** Socio-demographic and admission characteristics of children with SAM enrolled in the stabilizing center in Pawe General hospital (*N* = 760)

Variables	Categories	Frequency	Percent
Age	6–24 month	458	60.26
	25–48 month	219	28.82
	48–60 month	83	10.92
Sex	Male	333	43.82
	Female	427	56.18
Resident	Rural	556	73.16
	Urban	204	26.84
Maternal education status	Formally educated	516	67.89
	Had no formal education	244	32.11
Anemia	≥ 10 mg/dl	511	67.24
	< 10 mg/dl	249	32.76
Nasogastric intubation	Yes	318	41.84
	No	442	58.16
Vomiting	Yes	539	70.92
	No	221	29.08
Diarrheas	Yes	409	53.82
	No	351	46.18
Types of malnutrition	Marasmus	431	56.71
	Marasmus-kwashiorkor	230	30.26
	Kwashiorkor	99	13.03
Blood transfusion	Yes	168	22.11
	No	592	77.89
Breastfeeding	Yes	465	61.18
	No	295	38.82
Edema grading	No	425	55.92
	Grade+	277	36.45
	Grade++	40	5.26
	Grade+++	18	2.37
Vitamin A supplementation	Yes	584	76.84
	No	176	23.16
Folic acids supplementation	Yes	525	69.08
	No	235	30.92
MUAC	≤ 115 mm	131	17.24
	> 115 mm	629	82.76
Pneumonia	Yes	452	59.47
	No	308	40.53
TB infection	Yes	45	5.92
	No	715	94.08
Number of families	≤ 2	243	31.97
	3–4	419	55.13
	≥ 5	98	12.89
Admission types	New	685	90.13
	Readmission	75	9.8
Vaccination status	Completed	572	75.26
	Not completed	188	24.74
HIV infection	Positive	42	5.53
	Negative	718	94.47
Zink supplementation	Yes	349	45.9
	No	411	54.08
Treatment Outcome status	Cured	629	82.7
	Died	71	9.1
	Lost from follow up	39	5.3
	Medical transferred	21	2.7

### Baseline clinical and comorbidity characteristics

The majority 452 (59.47%) of participant cases had severe pneumonia at admission. The largest proportion of 718 (94.47%) respondents were negative for the HIV test. Moreover, 409 (53.82%) cases had diarrhea, but only 5.92% of admitted children had TB. During the first admission, 335 (44⋅1%) had edema and the mean weight of children during admission was 7.94 (± 2⋅8) kg. Similarly, the overall mean (± SD) of MUAC at first admission was found 11⋅2 (± 5.4) cm (Table 1).

### Time to relapse of SAM

The five years retrospective follow-up (N = 760) of participant observations yielded 6289 days of risk observation. The mean time to relapse of SAM was found to be 30.4(± 21.39) weeks. The dates of discharge from the first admission with minimum and maximum time were found to be 3 and 79 days, respectively. The average time (± SD) for treatment recovery from the first admission of the SAM case was calculated as 28.8(± 18.7) days. At the end of the follow-up period, 629 (82.7%) cases were cured, and the following 71(9.1%) were died, 39(5.3%) lost to follow-up, and 21 (2.7%) medically transferred to other hospitals. The overall incidence density rate of relapse was 10.8% (95% CI 8.3; 12.6) post-treatment discharged.

### Kaplan–Meier survival of SAM

**Fig. 1:**
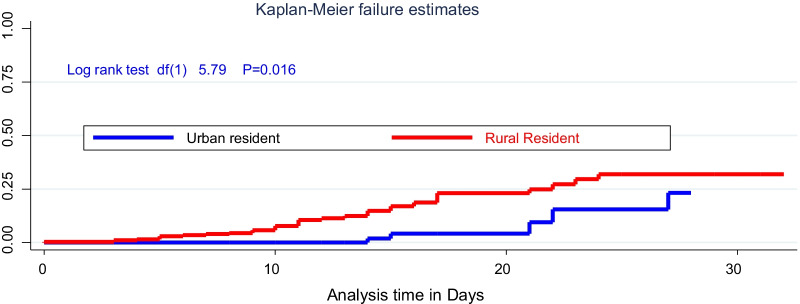
The hazard of time to relapse of SAM for under-five children stratified by the resident in Pawe General Hospital

**Fig. 2:**
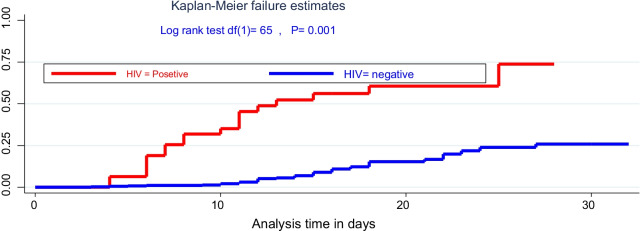
The hazard of time to relapse of SAM for under-five children stratified by HIV-infection status in Pawe General Hospital

There was a significant difference in time of relapsing for rural and urban SAM admitted cases, being rural residents, children were more quickly relapsed with a significant difference on the log-rank test (*χ*^2^; df(1) = 5.7, *p* = 0.016). Likewise, there was a significant survival difference time to relapse of SAM among HIV infected children with (*χ*^2^: (df = (1) 65, *p* = 0.001) as compared with their HIV-negative peers (Figs. 1, 2).

### Risk factors for time to relapse

During bivariable *Cox regression* analysis, variables were checked whether they were factors associated with time to relapse/re-admission of SAM at *p* value < 0.2, and selected for a candidate transferees of multivariable *Cox regression.* Finally**,** after adjusting confounding, eight variables were fitted to the final model with three predictors found as risk factors for time to relapse. Of those, children resident in a rural setting was a 5⋅2 time increase hazard of SAM relapse (AHR 5⋅3 = 95% CI 2⋅95, 13⋅87, *p* = 0.021) as compared with children living in urban settings. Likewise, the hazard of relapse was 6 times higher for children who had HIV infection (AHR6⋅8 = 95%CI; 4.1–11.9, *p* = 0.001) during the first admission as compared with HIV-negative children. Moreover, the risk of readmission for children admitted with edema was 3.5 (AHR = 3.5 = 95%CI; 1.92, 6.2, *p* = 0.018) times the increased risk of relapse as compared with non-edematous children admitted cases.

## Discussion

Our main finding was that children discharged as cured of SAM inpatient treatment remain persistent and at excess risk of relapse. We found out that the mean (± SD) time for relapse of SAM was found 30.4 (± 21.39) weeks, which is a longer relapse time compared to the report from 22 weeks [4] in Hadiya in Ethiopia, and [16] weeks in Nigeria [3]. This may be due to differences in study design and population as a study conducted in Nigeria was a prospective cohort conducted for only 6 months, while the present study captured data over 5 years of the retrospective cohort, which may result in differences. In the present study, the overall relapse rate of SAM was found to be10.8% (95% CI 8.3; 12.6) which is consistent with the finding in the Hadya zone, Ethiopia [4, 5]. This may show the commonality of the problem in our country, where the home environment is not usually altered although the child is treated for SAM in the health facility, which could lead to relapse of SAM. In some instants for SAM cases treated at home, there could be also sharing of the Ready-to-Use Therapeutic Food (RTUF) resulting in suboptimal treatment for foot step children and possible risks for cases to relapse. This should call the home environment specifically caregivers' awareness about the underlying risk factors [17]. Conversely, the finding of this report disagrees and is lower than 34.5% finding at south Gondar [18] and systematic review report in North Carolina [9]. The possible reason for the variations in this report is due to the difference in the underlying comorbidities, the caring practice of health care providers, health facility setup, and variation in the socioeconomic status of the population in these different study areas.

Regarding multivariable analyses, categorical variables that were independently associated with the risk of relapse of SAM were; being rural residents, edema during the first admission, and having HIV infection. In line with the previous findings, in different parts of the country [19, 20], and in Malawi [21], our report of SAM children presented with HIV infection were significantly associated with readmission, and required a long length of hospital stay as compared with their negative peers do have. In fact, both malnutrition and HIV have complex and multidirectional relationships that cause progressive immune system damage, with a synergistic effects, for instant malnutrition causes viral replication and hastens the progression of HIV disease by altering *β*-cell responses of hypersensitivity. On the other hand, HIV/AIDS accelerates the progression of immune impairment and repeated hospitalization through increasing loss of aptitude and through hastening of microbial translocation (16sDNA), intestinal damage (iFABP), and increased proteolysis of normal cells in HIV-positive children [22],which finally causes nutritional derangement and associated mala-absorptions.

Findings in our report indicated that the risk of readmission for admission with edema at first were 3.5 (AHR = 3.5 = 95%CI; 1.92, 6.2, *p* = 0.018) times the increased risk of relapse as compared with non-edematous children admitted cases. This finding is similar to the report of a study conducted in Hadya, Ethiopia [13, 23], and Metekl, Pawe hospitals [22]. Possibly early discharge form an inpatient treatment programs by using weight as discharge criteria from the treatment centers; they may have early weight gain as a remnant of nutritional edema-related weight. As nutritional edema affects the function of the glycocalyx is dependent upon sulfated proteoglycans and other glycosaminoglycans fundamentally related to a defect in sulphur metabolism, which can explain all clinical features of the condition, including the re-formation of edema [2, 5, 15]. The probability of getting readmission was increased for cases being a member of rural residents and the finding in our report show that being children resident in a rural setting were 5.3(AHR 5⋅3 = 95% CI 2⋅95, 13⋅87, *p* = 0.021) times the increased the hazard of SAM relapse as compared with their peers do live in an urban setting. Possibly the treatment discontinuation as defaulting due to several individuals compliant may like having more family members in the home, increase intention to be lost to follow-up and associated with earlier self-discharge possibly as this may cause readmission and nutritional derangements.

### Limitations of the study

Even though the strength of this paper comes from its study design (cohort), it was based on patients' secondary data, in which incompleteness was observed to some extent and lacked control over the quality of measurements taken during hospitalization. It was also impossible to analyze the socio-economic characteristics of caregivers and factors related to patient treatment could have influenced the outcome variable in a desirable or undesirable (Additional files 1 and 2).

## Conclusion

Nearly one in every ten severely acute malnourished under-five children relapsed within a mean time to relapse 30.4 (± 21.39) weeks posttreatment discharge. Time to relapse was significantly associated with being a rural resident for children, having edema during the first admission, and being HIV-infected cases. A protocol ought to be drafted for extending Supplementary Nutrition in Acute Malnutrition management program following discharge is highly needed.

## Supplementary Information

**Additional file 1**. English version checklists for data collections of SAM re-admissions.

**Additional file 2**. STATA (SE) version R/14; Data set for SAM re-admssions.

## Data Availability

The datasets employed in the current study are available from the corresponding author upon reasonable request via email.

## Abbreviations

AHR: Adjusted hazard ratio
CHR: Crude hazard ratio
CI: Confidence interval
FMOH: Ethiopian Federal Ministry of Health
MUAC: Mid-upper arm circumference
SAM: Severe acute malnutrition
NGT: Nasogastric intubation for feeding
WFH: Weight for height
SD: Standard deviation

## References

[1] FMOH. Federal democratic Republic of Ethiopia National Nutrition Program 2016–2020. Manuel 2020; Accessed 21 Dec 2021.

[2] Daures M, Phelan K, Issoufou M, Sawadogo O, Akpakpo B, Rather M (2021). Incidence of relapse following a new approach to simplifying and optimising acute malnutrition treatment in children aged 6–59 months: A prospective cohort in rural Northern Burkina Faso. J Nutr Sci.

[3] Adegoke O, Arif S, Bahwere P, Harb J, Hug J, Jasper P, Mudzongo P, Nanama S, Olisenekwu G, Visram A (2021). Incidence of severe acute malnutrition after treatment: A prospective matched cohort study in Sokoto, Nigeria. Matern Child Nutr.

[4] Lambebo A, Tamiru D, Belachew T (2021). Time to relapse of severe acute malnutrition and risk factors among under-five children treated in the health posts of Hadiya Zone, Southern Ethiopia. J Nutr Sci.

[5] Lambebo A, Temiru D, Belachew T (2021). Frequency of relapse for severe acute malnutrition and associated factors among under-five children admitted to health facilities in Hadiya Zone, South Ethiopia. PLoS ONE.

[6] Mulugeta DB (2020). Rural children remain more at risk of acute malnutrition following exit from community-based management of acute malnutrition program in South Gondar Zone Amhara Region, Ethiopia: a comparative cross-sectional study. PeerJ.

[7] Kebede F, Eticha N, Negese B, Giza M, Tolossa T, Wakuma B (2021). Predictors for a cure rate of severe acute malnutrition 6–59 month children in stabilizing center at Pawe General Hospital, Northwest Ethiopia: retrospective cohort study. Int J Child Health Nutr.

[8] Kebede F, Kebede T, Negese B, Abera A, Fentaw G, Kasaw A (2022). Incidence and predictors of severe acute malnutrition mortality in children aged 6–59 months admitted at Pawe General Hospital, Northwest Ethiopia. PLoS ONE.

[9] Stobaugh HC, Mayberry A, McGrath M, Bahwere P, Zagre NM, Manary MJ, Black R, Lelijveld N (2019). Relapse after severe acute malnutrition: a systematic literature review and secondary data analysis. Matern Child Nutr.

[10] Isanaka S, Grais RF, Briend A, Checchi F (2011). Estimates of the duration of untreated acute malnutrition in children from Niger, practice of epidemiology. Am J Epidemiol.

[11] Gomes-Neto AW, van Vliet IMY, Osté MCJ, de Jong MFC, Bakker SJL, Jager-Wittenaar H (2021). Malnutrition Universal Screening Tool and Patient-Generated Subjective Global Assessment Short Form and their predictive validity in hospitalized patients. Clin Nutr ESPEN.

[12] Kebede F, Kebede T, Kebede B, Abate A, Jara D, Negese B (2021). Time to develop and predictors for incidence of tuberculosis among children receiving antiretroviral therapy. Tuberc Res Treat.

[13] Cruz PLM, Soares BLM, da Silva JE, LimaESilva RR (2022). Clinical and nutritional predictors of hospital readmission within 30 days. Eur J Clin Nutr.

[14] Chang CY, Trehan I, Wang RJ, Thakwalakwa C, Maleta K, Deitchler M, Manary MJ (2013). Children successfully treated for moderate acute malnutrition remain at risk for malnutrition and death in the subsequent year after recovery. J Nutr.

[15] Dale NM, Salim L, Lenters L, Sadruddin S, Myatt M, Zlotkin SH (2018). Recovery and relapse from severe acute malnutrition after treatment: a prospective, observational cohort trial in Pakistan. Public Health Nutr.

[16] Lambebo A, Tamiru D, Belachew T (2021). Time-to-relapse-of-severe-acute-malnutrition-and-risk-factors-among-under-five-children-treated-in-the-health-posts-of-hadiya-zone-southern-ethiopia. J Nutr Sci.

[17] Deutz NE, Matheson EM, Matarese LE, Luo M, Baggs GE, Nelson JL (2016). Readmission and mortality in malnourished, older, hospitalized adults treated with a specialized oral nutritional supplement: a randomized clinical trial. Clin Nutr.

[18] Abitew DB, Yalew AW, Bezabih AM, Bazzano AN (2020). Predictors of relapse of acute malnutrition following exit from community-based management program in Amhara region, Northwest Ethiopia: An unmatched case-control study. PLoS ONE.

[19] Mena MB, Dedefo MG, Billoro BB (2018). Treatment outcome of severe acute malnutrition and its determinants among pediatric patients in West Ethiopia. Int J Pediatr.

[20] Tesfay W, Abay M, Hintsa S, Zafu T (2020). Length of stay to recover from severe acute malnutrition and associated factors among under-five years children admitted to public hospitals in Aksum, Ethiopia. PLoS ONE.

[21] Trehan I, Goldbach HS, LaGrone LN, Meuli GJ, Wang RJ, Maleta KM (2016). Antibiotics as part of the management of severe acute malnutrition. Malawi Med J.

[22] Molla M, Kebede F, Kebede T, Haile A (2022). Effects of Undernutrition and Predictors on the Survival Status of HIV-Positive Children after starting Antiretroviral Therapy (ART) in Northwest Ethiopia. Int J Pediatr.

[23] Bisrat G, Kulkarni U, Mariam YG (2017). Assessment of the nutritional status and associated factors of orphans and vulnerable preschool children with care and support from nongovernmental organizations in Hawassa Town. Global J Med Res.

