# Supplementary material for: Time to readmission and associated factors after post treatment discharge of severe acute malnourished under-five children in Pawe General Hospital
Source: J Health Popul Nutr. 2022 Jul 8;41:29. doi: 10.1186/s41043-022-00308-8 (PMC9270764; doi:10.1186/s41043-022-00308-8)
Supplement: Supplementary file 2 — Additional file 2. STATA (SE) version R/14; Data set for SAM re-admssions. [file 41043_2022_308_MOESM2_ESM.docx]

Annex 2: English versions of checklists for Incidence and predictors for time to lose after severe acute malnutrition (SAM) treatment started among under-five children admitted to Pawe general Hospital

**Principal Investigator:**

Fassikaw Kebede (BSC, MPH) ^1^*

**FK**: [**fassikaw123@gmail.com**](mailto:fassikaw123@gmail.com)

Date of data collection------------

Respondent’s signature------------

Data collectors ---------------------

Interview code ---------------------

Interview data----------------------

**PART (1): Checklist for socio-demographic questionnaire for quarantined individuals 2021**

| AT ADMISSION | Stabilization phase (week1-3) | | | |  | |
| --- | --- | --- | --- | --- | --- | --- |
| Unique SAM children Card No. | ---------- | | | |  | |
| Admission weight | -----------Kg | | | |  | |
| Admission height | -----------CM | | | |  | |
| Admission MUAC | --------- CM | | | |  | |
| Appetite test | yes | | | | No | |
| Discharge weight | --------------- | | | |  | |
| Discharged height | -------------- | | | |  | |
| Discharge MAUC | ----------------- | | | |  | |
| Age of children | ---------- | | | |  | |
| Admission date | -------//-----//------ | | | |  | |
| Discharge date /last treatment | -------//-----//--- | | | |  | |
| Sex of children | Male | | | | Female | |
| Resident of care giver | Urban | | | | Rural | |
| Religions of caregiver | --------------- | | | |  | |
| Permanent address of Woreda | ---------------- | | | |  | |
| Kebele | ------------- | | | |  | |
| Vaccination status of children | Fully vaccinated,----------  Not updated/defaulted, ------------  Not recorded ------------------ | | | |  | |
| Diagnosed SAM types | ---------------------------------- | | | | | |
| Baseline skin dermatitis | Present | | Absent | | | |
| Admission vomiting status | Present | | Absent | | | |
| Admission Nasogastric intubation | Present | | Absent | | | |
| Admission body temperature | Hyperemia (------) | | | Hyperthermia (------) | | |
| Anemia | Present | | | Absent | | |
| The phase of Rehabilitation  ( week1-3 week) | The phase of stabilizing  (3-5 week) | | | | | |
| Infection controlling |  |  | | | | |
| Diarrhea | Present | Absent | | | | |
| Pneumonia | Present | Absent | | | | |
| Skin dermatitis | Present | Absent | | | | |
|  |  |  | | | | |
| Edema status | Present | Absent | | | | |
|  | If present grading (+) , (++) , (+++) |  |  |  |  |  |
| IV fluid | given | Not given | | | | |
| Breastfeeding | yes breastfeed | No breastfeed | | | | |
| Admission type | New admission | Re-admission | | | | |
| **Vitamin A** | Given and recorded | Not given/ not mentioned | | | | |
| **Deworming** | Given and recorded | Not given/ not mentioned | | | | |
| **Amoxicillin as prophylaxis** | Given and recorded | Not given/ not mentioned | | | | |
| **Anti-malaria screening and prophylaxis** | Screened and given | Not given/ not mentioned | | | | |
| **IV Antibiotics/Parenteral antibiotics** | Given | Not given/ recorded | | | | |
| **Lists of Given Antibiotics** | Ampicillin and Gentamicin, Ampicillin or Gentamicin  Ceftriaxone, Metronidazole, Others | | | | | |
| **Blood file for malaria screening** | Screened and negative  Screened and positive | | | | | Not screened |
| **Folic acid** | Given | | | | | Not given |
| F-75 milk | Completed by phases | | | | | Not started or aborted |
| F-100 milk | Completed by phases | | | | | Not started or aborted |
| Tuberculosis screening | Yes screened negative  Yes screened and (+) | | | | | Not screened /unknown |
| **HIV tested** | Yes negative, Yes positive | | | | | Not tested/unknown |
| **Ascites and other medical complication** | Present and treated | | | | | No diagnosed /Absent |
| **Treatment Outcome of SAM admitted children** | 1. cured 2. death reported 3. Declared transferred out 4. Lost from SAM treatment center 5. Transferred out | | | | |  |
